# Perceptions and attitudes of nurse practitioners toward artificial intelligence adoption in health care

**DOI:** 10.1002/hsr2.70006

**Published:** 2024-08-21

**Authors:** Moustaq Karim Khan Rony, Sharker Md. Numan, Fateha tuj Johra, Khadiza Akter, Fazila Akter, Mitun Debnath, Sujit Mondal, Md. Wahiduzzaman, Mousumi Das, Mohammad Ullah, Mohammad Habibur Rahman, Shuvashish Das Bala, Mst. Rina Parvin

**Affiliations:** ^1^ Master of Public Health Bangladesh Open University Gazipur Bangladesh; ^2^ School of Science and Technology Bangladesh Open University Gazipur Bangladesh; ^3^ Masters in Disaster Management University of Dhaka Dhaka Bangladesh; ^4^ Master of Public Health Daffodil International University Dhaka Bangladesh; ^5^ Dhaka Nursing College affiliated with the University of Dhaka Dhaka Bangladesh; ^6^ Master of Public Health National Institute of Preventive and Social Medicine Dhaka Bangladesh; ^7^ Master of Science in Nursing National Institute of Advanced Nursing Education and Research Mugda Dhaka Bangladesh; ^8^ School of Medical Sciences Shahjalal University of Science and Technology Sylhet Bangladesh; ^9^ Master of Public Health Leading University Sylhet Bangladesh; ^10^ College of Nursing International University of Business Agriculture and Technology Dhaka Bangladesh; ^11^ Bangladesh Army (AFNS Officer) Combined Military Hospital Dhaka Dhaka Bangladesh

**Keywords:** artificial intelligence, attitudes, health care, nurse practitioners, perceptions

## Abstract

**Background:**

With the ever‐increasing integration of artificial intelligence (AI) into health care, it becomes imperative to gain an in‐depth understanding of how health care professionals, specifically nurse practitioners, perceive and approach this transformative technology.

**Objectives:**

This study aimed to gain insights into nurse practitioners' perceptions and attitudes toward AI adoption in health care.

**Methods:**

This qualitative research employed a descriptive and phenomenological approach using in‐depth interviews. Data were collected through a semi‐structured questionnaire with 37 nurse practitioners selected through purposive sampling, specifically Maximum Variation Sampling and Expert Sampling techniques, to ensure diversity in characteristics. Trustworthiness of the research was maintained through member checking and peer debriefing. Thematic analysis was employed to uncover recurring themes and patterns in the data.

**Results:**

The thematic analysis revealed nine main themes that encapsulated nurse practitioners' perceptions and attitudes toward AI adoption in health care. These included nurse practitioners' perceptions of AI implementation, attitudes toward AI adoption, patient‐centered care and AI, quality of health care delivery and AI, ethical and regulatory aspects of AI, education and training needs, collaboration and interdisciplinary relationships, obstacles in integrating AI, and AI and health care policy. While this study found that nurse practitioners held a wide range of perspectives, with many viewings AI as a tool to enhance patient care.

**Conclusions:**

This research provides a valuable contribution to the evolving discourse surrounding AI adoption in health care. The findings underscore the necessity for comprehensive education and training in AI, accompanied by clear and robust ethical and regulatory guidelines to ensure the responsible integration of AI in health care practice. Furthermore, fostering collaboration and interdisciplinary relationships is pivotal for the successful incorporation of AI in health care. Policymakers should also address the challenges and opportunities that AI presents in the health care sector. This study enhances the ongoing conversation on AI adoption in health care by shedding light on the perspectives of nurses, thereby shaping future strategies for AI integration.

## 1 INTRODUCTION

Artificial Intelligence (AI) represents the pinnacle of technological innovation, enabling machines to perform tasks that typically require human intelligence, such as learning and decision‐making.^1^ By utilizing advanced algorithms and data processing, AI systems can swiftly analyze large volumes of data to derive meaningful insights, adapting and improving their performance over time.^2^, ^3^ This technology has applications across diverse fields, from natural language processing to machine learning, and is transforming industries and daily life.^4^, ^5^

In nursing, AI demonstrates significant potential for enhancing patient outcomes through predictive analytics, personalized treatment plans, and early health deterioration detection.^6^ AI‐enhanced electronic health records (EHRs) improve the efficiency of patient data management, reducing documentation time and allowing nurses to concentrate more on patient care.^7^ Moreover, AI's integration into nursing education via simulation and virtual reality shows promise in advancing clinical skills and decision‐making.^8^ AI also plays a role in nursing research by automating literature reviews and thematic analyses, which enhances research efficiency and accuracy.^9^

In health care, AI is revolutionizing medical data processing, diagnostics, and treatment administration.^10^ Machine learning algorithms within AI systems improve clinical decision‐making, predict patient outcomes,^11^ and automate administrative tasks.^12^, ^13^ These technologies address persistent health care challenges such as diagnostic errors and treatment optimization.^14^ AI‐integrated EHRs support medical data analysis and predictive analytics,^15^ while telemedicine platforms use AI tools like chatbots and virtual health assistants to streamline patient communication and aid in diagnosing common conditions.^16^, ^17^ The rapid advancement of AI tools, including diagnostic aids and robotic surgical systems, is reshaping the health care landscape.^18^

Nurse practitioners are vital to health care delivery, serving as integral members of interdisciplinary teams and often providing primary care services.^19^, ^20^ They handle patient assessments, diagnoses, treatments, and chronic condition management.^21^ Besides their clinical duties, nurse practitioners educate and mentor, ensuring holistic and patient‐centered care.^22^ Their extensive patient interactions provide deep insights into patient needs and preferences,^23^ and they bridge the gap between patients and health care professionals, translating advancements like AI into practical care.^24^ Thus, their attitudes towards AI are crucial for the successful integration of these technologies in clinical settings.^25^

Globally, international collaboration is essential for advancing AI in health care. Sharing resources and knowledge accelerates the development of medical AI applications.^26^ Ethical guidelines from international organizations aim to address issues of bias, transparency, and privacy in AI development.^27^ Investments in education and the development of regulatory frameworks are necessary to balance innovation with safeguards.^28^, ^29^ National strategies, such as those in Bangladesh's High‐Tech Park, emphasize advanced infrastructure and international partnerships to drive health care AI innovation.^30^, ^31^

However, AI adoption faces challenges, including data privacy concerns, system interoperability issues, and resistance from health care professionals.^32^, ^33^ Understanding health care professionals' perspectives on AI is crucial, yet there is limited research in this area.^34^, ^35^ Insights into their attitudes can help address challenges and enhance AI integration, affecting patient care quality and trust in AI technologies.^36^, ^37^, ^38^ Ultimately, this study aimed to shed light on nurse practitioners' perceptions and attitudes regarding the adoption of AI in health care, exploring how these professionals perceive the evolving health care technology landscape and its impact on their practice.

## 2 METHODS

### 2.1 Research paradigm

In this study, the research paradigm was grounded in the constructivist paradigm.^39^ This paradigm acknowledged that individuals actively constructed their own realities and that their understanding of these realities was inherently subjective.^40^ Within this paradigm, the focus had been on the diverse perspectives and interpretations of the participants, which proved particularly relevant when exploring their perceptions and attitudes towards the incorporation of AI in health care.^41^ The study followed the guidelines of the Consolidated Criteria for Reporting Qualitative Research by Tong et al. ensuring rigorous reporting and transparency in qualitative research methodology.^42^

### 2.2 Theoretical framework

This study utilized the Technology Acceptance Model (TAM) as its theoretical framework.^43^ TAM is a widely used model that helps in understanding how users come to accept and use a technology. It posits that perceived usefulness and perceived ease of use are primary factors that influence users' attitudes towards adopting new technologies.^8^ By applying TAM, the study aimed to systematically explore the factors influencing nurse practitioners' perceptions and attitudes towards AI adoption in health care settings. This framework provided a structured approach to analyze the data, offering insights into the potential facilitators and barriers to AI adoption from the perspective of nurse practitioners.

### 2.3 Research approach

An exploratory, descriptive, and phenomenological research approach was employed in the study.^44^ This approach enabled a comprehensive examination of the lived experiences and perceptions^45^ of nurse practitioners regarding AI in health care. The exploratory aspect had been essential in uncovering the diversity of perceptions and experiences, while the descriptive nature ensured a detailed account of these experiences.^46^ The phenomenological approach had delved deep into the essence of these experiences, shedding light on the underlying meanings that nurse practitioners attached to AI adoption.

### 2.4 Data collection

The data were primarily collected between June 10 and July 25, 2023, through semi‐structured questions. This implies that the participants were given a certain degree of flexibility in responding while ensuring that there was a basic structure or a set of predefined questions guiding the interviews.^47^ The interviews took place face‐to‐face or via video conferencing for 50−60 min, depending on participant preference and availability. A semi‐structured format allowed for open‐ended questions while ensuring consistency in data collection.^48^ The interview guide was developed based on a thorough literature review, piloted among five participants, and some questions were reworded based on participants' comments. The interviews were audio‐recorded with participant consent and transcribed verbatim for analysis. Audio recording ensures a reliable record of the conversations and allows for a more accurate analysis of the data.^49^ Transcribed verbatim means that the recorded conversations were converted into written text without any alteration.^50^ The interview questions included:

1. Could you describe your experiences with AI in health care and how it impacted your role as a nurse practitioner?
2. What were your initial thoughts and feelings about the integration of AI into your daily clinical practice?
3. In your opinion, what are the most significant advantages that AI can offer in health care, and how do you envision it improving patient care?
4. What concerns or challenges did you associate with the adoption of AI in health care, and how did you think these issues could be addressed?
5. How did you perceive the relationship between your clinical expertize and the use of AI? Did you think it enhanced or competed with your skills as a nurse practitioner?
6. Could you share any specific ethical considerations or dilemmas you encountered or foresaw in the context of AI utilization in health care?
7. What kind of support or training did you believe was necessary for nurse practitioners to effectively incorporate AI into their practice?
8. Were there particular areas or aspects of health care where you thought AI could make the most significant impact, and why?
9. How did you see the future of health care evolving with the increasing presence of AI, and what role did you think nurse practitioners would play in this changing landscape?

### 2.5 Participant selection

A purposive sampling technique, specifically Maximum Variation Sampling and Expert Sampling, was employed to ensure the selection of participants with diverse characteristics.^45^ The inclusion of participants with diverse characteristics such as age, gender, highest degree, marital status, working experience, and employee type (Table 1) suggests the use of Maximum Variation Sampling. This approach ensures a broad representation of perspectives among nurse practitioners, enriching the data by capturing a wide spectrum of viewpoints.^51^

**Table 1:** Characteristics of participants.

Code	Gender	Age	Marital status	Highest degree	Working experience (years)	Employee type
NP1	Female	31	Married	MSN	8	Permament
NP2	Female	33	Married	MSN, MPH	7	Contructual
NP3	Male	38	Married	PhD	12	Contructual
NP4	Female	32	Married	MSN	6	Permament
NP5	Female	30	Married	MSN	4	Permament
NP6	Female	37	Married	MSN, MPH	11	Contructual
NP7	Female	36	Unmarried	Mphill	10	Permament
NP8	Male	33.5	Married	MSN	7.5	Temporary
NP9	Female	29	Unmarried	MPH	3	Permament
NP10	Female	28	Unmarried	MSN	5	Permament
NP11	Male	41	Married	PhD	18	Contructual
NP12	Female	34	Married	MSS, MSN	11	Permament
NP13	Male	36	Married	MSN	13	Permament
NP14	Male	39.5	Undisclosed	Mphill	16	Temporary
NP15	Female	33	Married	MSN	10	Permament
NP16	Female	50	Married	PhD	27	Permament
NP17	Female	28.5	Unmarried	MSN	5	Permament
NP18	Female	35	Married	MSS, MSN	12	Permament
NP19	Male	32	Married	MSN	9	Contructual
NP20	Female	34.5	Married	Mphill	11	Permament
NP21	Female	43	Married	MSN	20	Permament
NP22	Female	41	Married	MPH	18	Temporary
NP23	Female	47	Married	PhD	24	Permament
NP24	Female	34	Married	MSN	21	Permament
NP25	Female	51	Separate	PhD	30	Permament
NP26	Female	30	Married	MSN	7	Temporary
NP27	Male	37	Married	MPH	14	Permament
NP28	Female	33	Married	MSN	12	Permament
NP29	Female	43	Married	Mphill	20	Contructual
NP30	Female	32.5	Married	MSN	9	Temporary
NP31	Female	29	Unmarried	MSN	6	Permament
NP32	Female	40	Married	Mphill	17	Contructual
NP33	Male	31	Married	MSN	8	Permament
NP34	Female	36	Unmarried	MSN	13	Permament
NP35	Female	49	Married	PhD	26	Permament
NP36	Female	42	Married	MSN	21	Permament
NP37	Female	40	Married	MSN	17	Permament

Similarly, the specified criteria for participant selection, including having a minimum of 5 years of clinical experience and possessing 3 months of clinical experience with AI or attending a minimum of three conferences on AI adoption in health care, indicate the use of Expert Sampling. This method targets individuals with specialized knowledge or expertize^52^ in the field of health care and AI adoption. By including participants who meet these criteria, the researcher ensures that the sample consists of individuals with relevant experience and insights, contributing to a more nuanced understanding of the subject matter. Additionally, recruitment was conducted through professional networks, health care organizations, and nursing associations. Fifty‐nine potential participants were contacted via email or phone calls to explain the study's purpose, obtain informed consent, and schedule interviews. Among them, 37 nurse practitioners from five tertiary‐level health care institutions in Dhaka, Bangladesh, were recruited.

### 2.6 Trustworthiness

Ensuring the credibility and transferability of the research findings was of utmost importance, prompting the implementation of essential strategies.^53^ These included member checking and peer debriefing, each serving a distinct purpose in fortifying the robustness of the study.^54^ Member checking emerged as a pivotal step in maintaining the accuracy and interpretive integrity of the findings.^55^ By affording participants the opportunity to scrutinize a summary of their interview responses, it enabled them to confirm the precision and interpretation of their contributions, thereby ensuring the faithful representation of their viewpoints in the final research findings.^56^ This meticulous validation process was integral to the overall credibility of the research. Complementing this, peer debriefing stood as another indispensable element.^57^ The research team, through routine engagements in discussions, aimed to deliberate on the findings and interpretations collaboratively. These tailored sessions were crafted to minimize potential biases and enhance the overall credibility of the analysis, leveraging the diverse perspectives within the team.^58^ In concert, member checking and peer debriefing formed a comprehensive framework, fortifying the trustworthiness and applicability of the research findings.

### 2.7 Thematic analysis

The qualitative data, gathered through in‐depth interviews, underwent a rigorous and structured process of thematic analysis to uncover recurring themes, patterns, and categories within the data set.^52^ This analytical journey unfolded through a series of sequential stages, each contributing to the overall understanding of nurse practitioners' perceptions and attitudes towards the adoption of AI in health care. At the outset, the data familiarization stage entailed a comprehensive review of the interview transcripts, involving multiple iterations to foster a profound grasp of the content.^59^ This meticulous approach enabled the research team to deeply immerse themselves in the data and glean rich insights from the participants' perspectives. Subsequently, the analysis evolved into generating initial codes (Tables 2, 3, 4, 5, 6). During this phase, segments of text directly pertinent to the research question were systematically coded, meticulously extracting and emphasizing key elements within the data set.^60^

**Table 2:** Main theme, sub‐themes, participants' quotes, and codes.

Main theme	Sub‐themes	Participants quotes	Codes
Nurse practitioners' perceptions of AI implementation	AI as a tool for diagnosis	“AI has become a remarkable ally in the diagnostic process. It can analyze vast datasets, identify patterns, and suggest potential diagnoses that complement our clinical judgment (**NP33**)” “Initially, I was skeptical about AI's role in diagnosis, but now I see it as a valuable tool. It helps us confirm our suspicions, consider rare conditions, and provide patients with more well‐informed decisions (**NP11**)” “AI has the potential to reduce diagnostic errors by serving as a second pair of eyes. It helps us cross‐reference patient data and ensure we don't miss crucial details in complex cases (**NP7**)” “AI doesn't replace the human touch; it enhances it. It provides us with insights and information we might not have considered, ultimately improving our diagnostic accuracy (**NP3**)” “AI has shifted our diagnostic process from intuition to evidence‐based decision‐making. It's like having an experienced colleague who always has the latest research at their fingertips (**NP20**)” “As a diagnostic tool, AI streamlines our work and makes the process more efficient. It offers recommendations, but the final decision is still in our hands (**NP37**)” “AI isn't here to replace us; it's here to support us. It complements our expertize and helps us deliver better care by improving our diagnostic abilities (**NP23**)”	AI in the diagnostic process, Vast datasets analysis, Pattern identification, Potential diagnoses, Clinical judgment, Skepticism about AI, AI as a valuable tool, Confirming suspicions, Rare conditions consideration, Well‐informed decisions, Reducing diagnostic errors, Second pair of eyes, Cross‐referencing patient data, Not missing crucial details, Enhancing the human touch, Providing insights, Improving diagnostic accuracy, Shifting from intuition to evidence‐based decision‐making, Experienced colleague, Latest research access, Streamlining diagnostic work, Making the process more efficient, Offering recommendations, Final decision in the hands of humans, Supporting human expertize, Delivering better care, Improving diagnostic abilities
	AI in treatment decision‐making	“AI offers a wealth of treatment options. It suggests tailored interventions based on individual patient data, allowing us to make more personalized and effective treatment decisions (**NP35**)” “I used to think AI would undermine our treatment expertize, but now I see it as a powerful tool. It complements our clinical judgment by providing insights into treatment options we might have overlooked (**NP27**)” “AI's impact on treatment decision‐making is remarkable. It assists us in weighing the benefits and risks of various interventions, aiding in shared decision‐making with patients (**NP12**)” “AI revolutionizes how we approach treatment decisions. It serves as a knowledgeable colleague, offering data‐driven recommendations that inform our clinical expertize (**NP16**)” “AI streamlines the treatment decision process, making it more efficient. While it suggests options, the final decision always rests with us as health care providers (**NP35**)”	AI in treatment options, Tailored interventions, Individual patient data, Personalized treatment decisions, Effective treatment decisions, Undermining treatment expertize, Complementing clinical judgment, Insights into treatment options, Benefits and risks assessment, Shared decision‐making, Remarkable impact of AI, Data‐driven recommendations, Knowledgeable colleague, Revolutionizing treatment decisions, Informing clinical expertize, Streamlining the treatment decision process, Efficiency in treatment decisions, Final decision with health care providers
	AI‐enhanced patient communication	“AI revolutionizes how we communicate with patients. It provides accessible information, answers to common questions, and aids in patient education, empowering individuals to take charge of their health (**NP17**)” “I was initially unsure about AI in patient communication, but now I see its value. It's like having a virtual health companion, guiding patients and ensuring they receive the right information when they need it (**NP25**)” “AI has a transformative impact on patient communication. It enhances our ability to provide timely responses, offer personalized recommendations, and address concerns effectively (**NP2**)” “In busy clinical settings, AI is a game‐changer for patient communication. It streamlines administrative tasks and helps us focus on meaningful interactions, improving the overall patient experience (**NP6**)”	Patient communication, Accessible information, Common questions, Patient education, Empowerment, Virtual health companion, Value, Transformative impact, Timely responses, Personalized recommendations, Addressing concerns, Busy clinical settings, Game‐changer, Streamlining, Administrative tasks, Meaningful interactions, Patient experience, Health care revolution, AI in health care, Health companion, Information accessibility, Patient empowerment, Transformative technology, Administrative efficiency, Improved patient care
	AI's impact on workflow	“AI has brought efficiency to our daily workflow. It takes care of routine tasks, from documentation to scheduling, freeing up our time to focus on patient care. It's like having a skilled assistant who ensures everything runs smoothly (**NP11**)” “The impact of AI on workflow cannot be overstated. It reduces administrative burdens and minimizes errors, allowing us to work more effectively. It feels like a breath of fresh air in our busy health care environment (**NP22**)” “AI has changed the way we manage our daily tasks. It streamlines processes, optimizes resource allocation, and ensures our workflow is more organized. It's like having a trusted partner who ensures we provide the best care possible to our patients (**NP1**)”	AI, Efficiency, Daily workflow, Routine tasks, Documentation, Scheduling, Time management, Patient care, Skilled assistant, Administrative burdens, Error reduction, Health care environment, Workflow optimization, Resource allocation, Organization, Trusted partner, Process streamlining, Task management, Resource optimization, Quality care, Workflow improvement, Health care efficiency, Administrative tasks, Error prevention, Patient‐focused care
	AI‐driven predictive analytics	“AI's predictive capabilities empower us to make data‐driven decisions. It's like having a crystal ball that guides us in tailoring care to individual patient needs (**NP18**)” “The impact of AI‐driven predictive analytics is undeniable. It streamlines care planning, reduces preventable hospitalizations, and enhances our ability to provide patient‐centered care (**NP13**)” “In health care, AI predictive analytics is a welcomed addition. It optimizes resource allocation, decreases health care costs, and, most importantly, leads to better patient experiences and outcomes (**NP6**)”	Predictive capabilities, Data‐driven decisions, Crystal ball, Tailoring care, Individual patient needs, AI‐driven predictive analytics, Care planning, Preventable hospitalizations, Patient‐centered care, Health care, Resource allocation, Health care costs, Patient experiences, Patient outcomes, Predictive modeling, Decision‐making, Health care optimization, Resource management, Health care efficiency, Health care improvements, Quality of care, Patient‐focused care, Data analysis
	AI and patient outcomes	“AI's contribution to patient outcomes is remarkable. It helps us make more accurate diagnoses, choose effective treatments, and closely monitor patient progress. It's like having a vigilant guardian, ensuring that we provide the best care for our patients (**NP16**)”“AI's influence on patient outcomes is undeniable. It allows us to practice more precision medicine, reduce complications, and enhance patient satisfaction. It's a valuable addition to our health care toolkit (**NP8**).	Patient outcomes, Accurate diagnoses, Effective treatments, Patient progress, Vigilant guardian, Precision medicine, Complications, Patient satisfaction, Health care toolkit, Medical decision‐making, Treatment selection, Patient monitoring, Health care quality, Health care improvement, Precision health care, Improved care, Medical advancements, AI applications, Health care technology
	Trust in AI decision support	“Trust in AI decision support has been hard‐earned. Initially, I was skeptical, but over time, I've come to rely on it. It's like a dependable assistant, offering suggestions that align with our expertize, improving patient care (**NP34**)” “AI's role in decision support is valuable, but it doesn't replace our judgment. It's like having a knowledgeable partner who provides insights, but the final decision rests with us (**NP20**)” “AI's place in decision support has earned our trust. It streamlines our decision‐making process, offering data‐driven insights that complement our expertize. It's become a reliable tool in our health care journe (**NP28**)”	Accurate diagnoses, Effective treatments, Patient progress, Vigilant guardian, Precision medicine, Complications, Patient satisfaction, Health care toolkit, Trust in AI, Decision support, Skepticism, Dependable assistant, Suggestions, Expertize, Improved patient care, Valuable role, Knowledgeable partner, Data‐driven insights, Final decision, Trustworthiness, Decision‐making process, Reliable tool, Health care journey

**Table 3:** Main theme, sub‐themes, participants' quotes, and codes.

Main theme	Sub‐themes	Participants quotes	Codes
Attitudes toward AI adoption	Enthusiasm for AI integration	“AI's impact on treatment decisions has been revolutionary. It's like having a personal assistant who crafts treatment plans tailored to each patient's unique data. Initially, we were skeptical, but now we see AI as an invaluable tool that enables personalized and effective decision‐making. It has truly changed the way we approach patient care (**NP7**)” “The integration of AI in patient communication is a game‐changer. It's like having a virtual health companion that answers common questions, provides accessible information, and enhances patient education. Our busy clinical setting greatly benefits from AI's ability to streamline administrative tasks, allowing us to focus on meaningful patient interactions. The patient experience has improved dramatically, and we're excited about the positive impact AI has had on health care delivery (**NP1**)” “We've come to trust AI as a dependable partner in our health care journey. At first, we were uncertain, but now we rely on AI as a knowledgeable assistant. It complements our clinical judgment, offering valuable insights and streamlining decision‐making. AI has earned its place as a reliable health care tool, aligning seamlessly with our expertize and ultimately enhancing patient care (**NP12**)”	Treatment decisions, Personal assistant, Treatment plans Skeptical, Invaluable tool, Personalized decision‐making, Patient care, Integration of AI, Patient communication, Virtual health companion, Accessible information, Patient education, Streamline administrative tasks, Meaningful patient interactions, Patient experience, Health care delivery, Dependable partner, Knowledgeable assistant, Clinical judgment, Valuable insights, Decision‐making, Reliable health care tool, Health care expertize, Enhanced patient care
	Concerns about AI's impact	“AI adoption in health care is promising, but I have concerns about potential dehumanization. It's like we're at risk of losing the personal touch in patient care. As nurse practitioners, we must strike a balance to ensure technology enhances, not replaces, the human element in health care (**NP14**)” “The security of patient data with AI is a significant concern. It's as if we're handing over sensitive information to machines. We need strong safeguards to protect patient privacy, making sure AI doesn't compromise the confidentiality and security of health care data (**NP3**)” “While AI can streamline tasks, I worry about potential job displacement. It's like we're creating a future where AI takes over, and our roles diminish. We hope that AI complements our work rather than replaces it, preserving our place in patient care (**NP19**)” “We must be vigilant about AI bias in health care. It's akin to a digital referee, but we need to ensure that it doesn't perpetuate biases present in the data it learns from. We're concerned that AI could inadvertently reinforce health care disparities and inequalities (**NP26**)”	AI adoption, Health care, Dehumanization, Personal touch, Patient care, Nurse practitioners, Technology, Balance, Security, Patient data, Safeguards, Patient privacy, Confidentiality, Security of health care data, Job displacement, Future, AI's role, Complement, Preserving roles, Vigilance, AI bias, Digital referee, Data learning, Biases, Health care disparities, Inequalities, Concerns, Potential issues, Technology impact, Patient well‐being
	Regulatory compliance	“As nurse practitioners, we're concerned about AI's impact on regulatory compliance. It's like navigating a complex maze; we need clear guidelines to ensure AI aligns with health care regulations and standards (**NP17**)” “AI's rapid integration in health care demands strict oversight. It's as if we're in uncharted territory, and the rules need to be well‐defined to protect patients and practitioners alike (**NP4**)” “The evolving AI landscape raises questions about accountability. It's like a shifting landscape; we need to establish clear responsibilities for AI in health care to ensure ethical and legal compliance (**NP9**)”	Nurse practitioners, AI impact, Regulatory compliance, Health care regulations, Guidelines, Rapid integration, Strict oversight, Uncharted territory, Well‐defined rules, Patient protection, Accountability, Ethical compliance, Legal compliance, AI landscape, Responsibilities, Clear responsibilities, Health care standards, Compliance issues, Health care ethics, Rule establishment, Ethical and legal considerations, Patient safety, AI in health care, Health care practitioners, Regulatory guidelines
	AI and health care inequality	“As health care providers, we must address the potential for AI to exacerbate health care inequality. It's like a double‐edged sword; we need to ensure that AI is implemented in a way that bridges disparities and doesn't leave vulnerable populations behind (**NP11**)” “AI's impact on health care inequality cannot be underestimated. It's as if we're at a crossroads, where our decisions about AI usage can either reduce or widen existing health care disparities (**NP21**)” “AI has the potential to revolutionize health care, but we must be vigilant. It's like a balancing act; we need to ensure AI benefits all, rather than creating a wider gap in health care access and outcomes (**NP6**)” “Addressing health care inequality is a moral imperative. It's as if we're at a critical juncture, where the responsible integration of AI can help level the playing field and ensure equitable health care access for all (**NP27**)”	Health care providers, Health care inequality, Disparities, Vulnerable populations, Crossroads, AI usage, Health care disparities, Revolutionize health care, Vigilant, Balancing act, Health care access, Outcomes, Moral imperative, Integration of AI, Level the playing field, Equitable health care access, Responsible integration, Health care outcomes, AI's potential impact, Bridging disparities, Vulnerable communities, Ethical considerations, Equity in health care, Reducing disparities, Ethical use of AI
	Nurse practitioners' willingness to adopt AI	“We see AI as a valuable tool. It's like a trusted colleague who can support us. We're willing to embrace AI to enhance efficiency and outcomes in health care (**NP18**)” “Nurse practitioners are ready for AI. It's like a new ally in health care. We're open to the opportunities it brings and are excited about its potential to elevate patient care (**NP23**)” “AI is an exciting addition to our practice. It's like a partner in progress. We're willing to adopt AI and work alongside it to provide better health care solutions (**NP13**)”	Valuable tool, Trusted colleague, Embrace AI, Enhance efficiency, Health care outcomes, Nurse practitioners, New ally, Opportunities, Elevate patient care, Exciting addition, Partner in progress, Adopt AI, Health care solutions, Collaboration, AI integration, Positive outlook, Support in health care, Potential benefits, Openness to AI, AI's role in health care, Patient care improvement, Progress in health care, Partnering with AI, Health care advancement

**Table 4:** Main themes, sub‐themes, participants' quotes, and codes.

Main themes	Sub‐themes	Participants quotes	Codes
Patient‐centered care and AI	Maintaining personal connection	“AI helps, but I always make sure to maintain that personal touch. Patients need to know we're here for them, not just relying on machines (**NP36**)” “AI can support us, but it can't replace the empathy and understanding we provide. That's what truly makes patient care special (**NP5**)” “Our connection with patients goes beyond AI. It's about the human touch, the reassurance, and the trust they have in us. (**NP22**)”	AI, Personal touch, Patient needs, Empathy, Understanding, Patient care, Support, Connection, Human touch, Reassurance, Trust, Health care, AI's role, Patient‐provider relationship, Patient‐centered care, Empathetic care, Human interaction, Trust in health care, Special care, AI limitations
	Balancing technology and human interaction	“It's a delicate balance – using AI to streamline tasks and ensure efficient care while also preserving meaningful human interactions. Patients appreciate the blend of technology and human touch (**NP27**)” “AI is a powerful ally, but we must use it wisely. We need to find that sweet spot where technology enhances our care without overshadowing our personal connections with patients (**NP13**)” “Patients want the benefits of technology, but they also value face‐to‐face interactions. It's our responsibility to strike the right balance (**NP25**)”	AI, Delicate balance, Streamline tasks, Efficient care, Meaningful human interactions, Technology and human touch, Powerful ally, Wise usage, Sweet spot, Personal connections, Patients’ preferences, Face‐to‐face interactions, Patient values, Technology benefits, Right balance, Health care delivery, Patient‐centered care, Technology integration, Human interaction, Care optimization
	Communication and explanation of AI use to patients	“Transparency is key when it comes to AI. Patients deserve to know how it's being used in their care and the benefits it brings (**NP33**)” “Explaining AI to patients in simple terms builds trust and reduces anxiety. They appreciate being part of the decision‐making process (**NP16**)” “Our role is not just to use AI but to communicate its role effectively to patients. It's about making them comfortable with technology in their healthcar (**NP37**)” “Patients are curious about AI, and we need to be open about it. A clear and honest discussion about its use helps them feel more engaged in their care (**NP2**)”	Transparency, AI usage, Patient awareness, Benefits of AI, Explanation, Trust‐building, Decision‐making process, Communication, Technology in health care, Patient comfort, Curiosity, Openness, Clear discussion, Honest communication, Patient engagement, Health care information, Patient‐centered care, Health care technology, Information sharing, AI awareness
	AI's role in patient engagement	“AI can actually enhance patient engagement. It helps us provide them with information and support more efficiently, making them feel more involved in their care (**NP11**)” “AI empowers patients by giving them easy access to information. It's a game‐changer in making them active participants in their health journey (**NP32**)” “The beauty of AI is that it can be a bridge to better patient engagement. It facilitates communication, which ultimately leads to more engaged and informed patients (**NP5**)”	AI, Patient engagement, Information, Support, Efficiency, Involvement, Empowerment, Access to information, Active participants, Health journey, Communication, Informed patients, Facilitation, Technology in health care, Patient‐centered care, Health care engagement, Information access, Active involvement, Better patient outcomes, AI's impact on engagement
Quality of health care delivery and AI	Efficiency and timeliness	“AI has revolutionized health care efficiency. It streamlines administrative tasks, allowing us to focus more on patients. Appointments, records, and communication have become swift and error‐free, improving overall health care quality (**NP33**)” “Efficiency is crucial for health care delivery, and AI delivers. It reduces time spent on paperwork, helping us see more patients without compromising care quality (**NP19**)” “With AI, we're not wasting precious minutes on administrative tasks. We can dedicate more time to patients, ensuring their needs are met promptly (**NP25**)”	AI, Health care efficiency, Administrative tasks, Focus on patients, Appointments, Records, Communication, Swift, Error‐free, Health care quality, Efficiency, Health care delivery, Paperwork, Patients, Care quality, Time management, Health care productivity, Patient care, Administrative efficiency, AI's impact, Time‐saving, Quality improvement, Health care processes, AI's role in health care, Workflow improvement
	Reduction in diagnostic errors	“AI complements our clinical judgment by minimizing diagnostic errors. It's like a safety net, reducing the likelihood of missed or incorrect diagnoses (**NP33**)” “The reduction in diagnostic errors is evident with AI. It's a critical addition to our health care toolkit, enhancing our confidence in providing accurate and timely diagnoses (**NP17**)” “AI's role in reducing diagnostic errors cannot be overstated. It's like having an experienced colleague who checks our work, leading to better patient care (**NP8**)”	AI, Clinical judgment, Diagnostic errors, Safety net, Missed diagnoses, Incorrect diagnoses, Health care toolkit, Confidence, Accurate diagnoses, Timely diagnoses, Experienced colleague, Better patient care, Error reduction, AI's role, Medical diagnosis, Health care improvement, Diagnostic accuracy, Patient safety, Health care technology, AI's impact on diagnosis
	Improved treatment plans	“Treatment plans are more precise and patient‐centric with AI. It helps us make decisions based on data, improving treatment outcomes (**NP3**)” “AI has transformed our approach to treatment planning. It considers all relevant factors, ensuring patients receive the most suitable and effective care (**NP34**)” “AI‐driven treatment planning takes patient care to the next level. It's like having a co‐pilot who guides us to make the best decisions for our patients (**NP10**)”	Treatment plans, AI, Precision, Patient‐centric, Data‐driven decisions, Treatment outcomes, Transformation, Approach, Relevant factors, Suitable care, Effective care, AI‐driven, Next level, Co‐pilot, Best decisions, Patient care, Health care improvement, Treatment effectiveness, AI's role in health care, Decision‐making, Personalized care, Treatment optimization, Health care quality
	AI‐enhanced care coordination	“Care coordination is smoother and more efficient with AI. It facilitates real‐time information sharing among health care providers, leading to better patient care (**NP14**)” “AI is the missing piece in care coordination. It simplifies the process, ensuring that every health care provider is on the same page, ultimately benefiting the patient (**NP25**)”	Care coordination, Smoother, Efficient, Real‐time information sharing, Health care providers, Better patient care, Missing piece, Process simplification, Same page, Patient benefit, Health care integration, Coordination effectiveness, Health care communication, AI's role, Health care teamwork, Collaboration, Information exchange, Patient‐centered care, Health care improvement
	AI‐driven continuous monitoring	“AI‐driven continuous monitoring ensures patients are under constant observation. It's a valuable safety net, alerting us to any anomalies and allowing for timely interventions (**NP36**)” “With AI, continuous monitoring is more comprehensive. It provides a safety net for patients, helping us identify potential issues before they escalate (**NP24**)”	AI‐driven, Continuous monitoring, Patients, Observation, Safety net, Anomalies, Timely interventions, Comprehensive, Identify potential issues, Health care technology, Patient safety, AI's role, Monitoring improvement, Health care monitoring, Early detection, Health care intervention, Patient care, Anomaly detection, Health care outcomes, Patient well‐being
	AI and preventive care	“AI has a significant role in preventive care. It analyzes patient data to identify potential risk factors, allowing us to take proactive measures and prevent health issues (**NP6**)” “Preventive care is enhanced with AI. It identifies patients at risk, allowing us to intervene early and promote healthier outcomes (**NP15**)” “AI acts as a preventive care partner. It's like a health detective, helping us identify risks and recommend measures to keep our patients healthy (**NP34**)”	Preventive care, Patient data, Risk factors, Proactive measures, Health issues, Prevention, Enhanced care, Patients at risk, Early intervention, Healthier outcomes, Preventive care partner, Health detective, Health recommendations, Patient wellness, Health care improvement, AI's role in health care, Risk assessment, Health promotion, Preventive measures

**Table 5:** Main themes, sub‐themes, participants' quotes, and codes.

Main themes	Sub‐themes	Participants quotes	Codes
Ethical and regulatory aspects of AI	Privacy and data security	“In the age of AI, data is a powerful currency, but it's also a vulnerability. We need to be vigilant in safeguarding it, implementing encryption, access controls, and audits to protect patient information. It's not just about technology; it's about ethics and trust (**NP10**)” “Patients entrust us with their most intimate information. As we integrate AI, we must be unwavering in our commitment to data security. Transparency in how we handle their data and strong encryption are Nonnegotiable (**NP3**)”	Data, AI (Artificial Intelligence), Currency, Vulnerability, Vigilant, Safeguarding, Encryption, Access controls, Audits, Patient information, Ethics, Trust, Intimate information, Integration, Data security, Transparency, Handling data, Strong encryption, Commitment, Nonnegotiable
	Informed consent	“Informed consent in the context of AI is a multifaceted challenge. Patients should not only understand how AI contributes to their care but also be informed about the implications and potential outcomes. We need comprehensive educational materials and conversations to empower patients to make informed choices (**NP25**)” “Informed consent with AI is a complex conversation. It's not just about the technology; it's about acknowledging patient values, preferences, and concerns. Our duty is to provide patients with the knowledge and choice to make decisions aligned with their wishes (**NP14**)”	Informed consent, Multifaceted, Patients, Understanding, Implications, Potential outcomes, Educational materials, Conversations, Empowerment, Choices, Patient values, Preferences, Concerns, Duty, Knowledge, Decisions, Wishes, Complex conversation
	Ensuring equity in AI use	“Equity matters in AI adoption. We must address biases to ensure fair treatment for all patients, regardless of their background (**NP31**)” “AI should enhance health care for everyone. We must actively work to eliminate biases and disparities in its application (**NP9**)” “AI has the potential to worsen disparities. We need to actively strive for fairness, ensuring equal access and outcomes for all (**NP24**)”	Equity, AI, Bias, Fair treatment, Patients, Background, Health care, Disparities, Application, Potential, Worsen, Strive, Fairness, Equal access, Outcomes
Education and training needs	Integration of AI in educational programs.	“AI should be an integral part of educational programs to prepare future health care professionals. We need courses that cover its basics, potential applications, and ethical considerations (**NP12**)” “Incorporating AI into education can bridge the gap between theory and practice. Students need hands‐on experience and exposure to AI tools and applications (**NP5**)” “Educational institutions must adapt their curricula to include AI content. AI is transforming health care, and students should graduate with AI proficiency, ready to meet evolving health care demands (**NP18**)”	Educational programs, Health care professionals, Courses, Basics, Potential applications, Ethical considerations, Education, Bridge the gap, Theory and practice, Students, Hands‐on experience, Exposure, Applications, Educational institutions, Curricula, AI content, Transforming health care, AI proficiency
	Training on AI implementation	“I think learning to use AI tools should be a part of our professional development. Training is vital to maximize the benefits AI can offer in our clinical work (**NP28**)” “AI training is an investment in better patient care. We need tailored programs that address the practical aspects of implementing AI in diverse health care settings (**NP2**)”	Learning, Professional development, Training, Benefits, Clinical work, Investment, Patient care, Tailored programs, Practical aspects, Implementing AI, Health care settings, Professional training
	AI training challenges	“AI training can be challenging due to the rapid pace of technological advancements. Continuous learning and staying up to date are essential for us to remain effective practitioners (**NP37**)” “One challenge in AI training is the need for customized content that addresses specific health care contexts. Generic AI courses may not meet our real‐world needs (**NP21**)” “AI training challenges include finding time and resources for learning while managing our clinical responsibilities. Institutions must provide support for practitioners to acquire the necessary skills (**NP32**)”	Training, Challenging, Technological advancements, Continuous learning, Staying up to date, Effective practitioners, Customized content, Health care contexts, Generic AI courses, Real‐world needs, Time, Resources, Clinical responsibilities, Institutions, Support, Necessary skills

**Table 6:** Main themes, sub‐themes, participants' quotes, and codes.

Main themes	Sub‐themes	Participants quotes	Codes
Collaboration and interdisciplinary relationships	Interactions with AI specialists	“Collaboration with AI specialists enhances our decision‐making. Their expertize complements our clinical knowledge, ensuring patients receive the best care (**NP1**)” “Working with AI specialists creates a valuable synergy. It's a partnership that leverages their technical skills and our patient‐centered approach for optimal outcomes (**NP9**)” “Interactions with AI specialists are enlightening. They introduce new possibilities and tools that can truly transform how we deliver care (**NP20**)”	Collaboration, AI specialists, Decision‐making, Expertize, Clinical, knowledge, Patients, Best care, Synergy, Partnership, Technical skills, Patient‐centered approach, Optimal outcomes, Interactions, Enlightening, Possibilities, Tools, Transform, Deliver care
	Nurse practitioners' input on AI implementation	“Incorporating nurse practitioners' insights into AI implementation is a smart move. We bridge the gap between technology and patient needs, creating a more patient‐centric AI solution (**NP15**)” “Our involvement in AI implementation is about making it more human. We bring the patient perspective to the table, ensuring AI serves health care's core mission—patient well‐being (**NP11**)”	Nurse practitioners, AI (Artificial Intelligence) implementation, Insights, Technology, Patient needs, Patient‐centric, Solution, Human, Patient perspective, Health care, Core mission, Patient well‐being, Involvement, Bridge the gap
Obstacles in integrating AI	Integration with existing systems	“Integrating AI with our current systems is a complex process. It requires thoughtful planning to avoid disruptions in our workflow (**NP4**)” “Our systems are deeply ingrained in our practice. Integrating AI needs to be smooth to ensure minimal disruptions to patient care (**NP29**)” “Integrating AI into our existing systems is like fitting puzzle pieces. It takes time and patience to create a coherent health care environment (**NP8**)”	Integrating AI, Current systems, Complex process, Thoughtful planning, Workflow, Practice, Disruptions, Patient care, Existing systems, Smooth integration, Puzzle pieces, Time, Patience, Health care environment
	Technical barriers and compatibility	“Technical barriers can be frustrating. Ensuring compatibility between AI systems and our existing technology is crucial to minimize hiccups (**NP19**)”“AI compatibility with our existing technology is a key concern. We need solutions that are seamless and user‐friendly to ensure successful integration (**NP30**)” “Technical challenges can impede AI integration. Compatibility and user‐friendliness are paramount to avoid disruptions in our daily practice (**NP27**)”	Technical barriers, Compatibility, AI (Artificial Intelligence) systems, Existing technology, Frustrating, Hiccups, Key concern, Solutions, Seamless, User‐friendly, Integration, Technical challenges, Disruptions, Daily practice
	Financial constraints	“Financial constraints are a real challenge in AI adoption. The costs associated with integration can be a barrier, but we must find cost‐effective solutions (**NP31**)” “Financial constraints make AI adoption difficult. We need to explore options that are economically viable to ensure successful integration (**NP17**)” “Budget limitations hinder AI adoption. Finding cost‐effective ways to implement AI is a priority to overcome financial constraints (**NP29**)” “AI integration can strain our budgets. We must identify financially sustainable paths for successful implementation (**NP6**)”	Financial constraints, AI (Artificial Intelligence) adoption, Costs, Integration, Barrier, Cost‐effective solutions, Budget limitations, Economically viable, Successful integration, Budgets, Economically sustainable, Implementation, Priority, Financially sustainable
AI and health care policy	Government regulations and AI	“AI's integration into health care calls for comprehensive government regulations. These regulations must strike a delicate balance, fostering innovation while upholding patient rights and safety. Policymakers should engage health care practitioners in continuous discussions to adapt to the evolving AI landscape (**NP26**)” “I believe government regulations play a pivotal role in guiding the ethical use of AI in health care. They must be agile and adaptive, capable of keeping pace with AI advancements. A strong collaboration between health care professionals and policymakers is crucial to ensure that AI is harnessed for the betterment of patient care (**NP7**)”	Health care, Government regulations, Patient rights, Safety, Policymakers, Health care practitioners, Innovation, Ethics, Continuous discussions, Evolving AI landscape, Ethical use, Agile, Adaptive, Collaboration, AI, advancements, Betterment, Patient care
	Health care institutions' AI policies	“AI policies within health care institutions are indispensable. They provide clear guidelines and establish a framework for the responsible use of AI technologies. These policies help in ensuring transparency and maintaining patient trust while fostering a culture of ethical AI adoption within the organization (**NP21**)” “Health care institutions require robust AI policies that set clear boundaries and ethical standards. These policies help maintain transparency and accountability, ensuring that AI benefits both patients and providers. They should evolve with technological advancements, offering guidance for ethical AI integration (**NP3**)”	Policies, Health care institutions, Guidelines, Framework, Responsible use, Technologies, Transparency, Patient trust, Ethical AI adoption, Organization, Boundaries, Ethical standards, Accountability, Patients, Providers, Technological advancements, Guidance, Integration
	Insurance and reimbursement with AI	“Reimbursement policies should reflect the responsible integration of AI in health care. These policies must incentivize health care providers to utilize AI for quality patient care. Fair compensation for AI adoption aligns with ethical AI practices and encourages its responsible use (**NP23**)” “AI's role in insurance and reimbursement needs to ensure that health care providers are fairly compensated for the use of AI. These policies should not only encourage the ethical adoption of AI but also benefit patients through improved health care outcomes (**NP32**)”	Reimbursement policies, Responsible integration, Health care, Incentivize, Health care providers, Quality patient care, Fair compensation, Ethical AI practices, Responsible use, Insurance, Benefit patients, Improved health care outcomes

**Figure 1:**
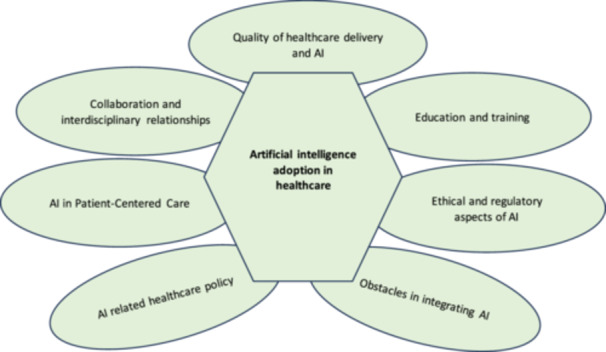
Nurse practitioners' perspectives regarding artificial intelligence adoption in health care.

The following stage of analysis revolved around theme development. Here, four researchers carefully identified initial codes and potential themes. Simultaneously, the analysis process involved an ongoing review and refinement of the identified themes, ensuring their accuracy and cohesiveness^61^ (Figure 1). Where necessary, sub‐themes were developed to capture nuanced aspects of the data comprehensively. In the final stage of this analytical journey, the research findings were meticulously presented in a clear and coherent manner, bolstered by direct quotes from the participants (Tables 2, 3, 4, 5, 6). This reporting phase marked the culmination of the analytical process, providing a solid foundation for understanding nurse practitioners' perceptions and attitudes regarding the integration of AI in health care.

### 2.8 Ethical considerations

This research rigorously adhered to ethical guidelines, ensuring the protection of participants' rights and well‐being.^62^ Participants received a comprehensive consent form outlining the study's objectives, procedures, and their rights, including the freedom to withdraw at any time without negative consequences. Anonymity and confidentiality were strictly maintained, with all data securely stored to prevent unauthorized access.^63^ After their participation, individuals were offered debriefing sessions to discuss their experiences and address any concerns.^64^ Ethical standards were upheld through approval from the Institutional Review Committee at Bangladesh Open University (approval number BOU/MPH/ACADEMIA/1301/05112023), ensuring the study met all ethical requirements and safeguarded participants' rights and safety throughout.

## 3 RESULTS

### 3.1 Nurse practitioners' perceptions of AI implementation

Nurse practitioners in the study showed a positive shift in their views on AI as a diagnostic tool, appreciating its ability to analyze data, recognize patterns, and support diagnoses. They saw AI as a complement to clinical judgment, enhancing workflow, diagnostic accuracy, and evidence‐based decisions. AI improved patient communication by offering accessible information and aiding shared decision‐making. Initially skeptical, practitioners now value AI for streamlining tasks, reducing administrative burdens, and improving patient care, highlighting its growing role in their health care toolkit (Table 2).

### 3.2 Attitudes toward AI adoption

Nurse practitioners in this study warmly embraced AI, viewing it as transformative for personalized treatment and patient care. They valued AI as a virtual health companion that improves communication and streamlines health care delivery. However, concerns about dehumanization, data security, and job displacement were noted. Practitioners emphasized AI should complement, not replace, their roles and called for vigilance against biases. They advocated for clear guidelines to align AI with health care regulations, ensuring ethical integration and addressing potential disparities. Despite these concerns, AI was welcomed as a valuable tool for enhancing health care solutions (Table 3).

### 3.3 Patient‐centered care and AI

Participants in the study stressed the importance of maintaining a personal connection in health care, highlighting the irreplaceable human touch, empathy, and trust provided by health care providers. While acknowledging AI's benefits, they emphasized that AI cannot replace these critical elements of patient care. The study underscored the need for a balance where AI enhances rather than overshadows the personal connection valued by patients. Nurse practitioners also highlighted the importance of transparent communication about AI with patients, advocating for clear, simple explanations and involving patients in decision‐making. This approach fosters trust, reduces anxiety, and empowers patients by making them active participants in their health care journey (Table 4).

### 3.4 Quality of health care delivery and AI

Nurse practitioners praised AI for its transformative impact on health care efficiency. They noted AI's ability to streamline administrative tasks, such as appointments and record‐keeping, leading to error‐free operations and more time for patient care. AI also significantly reduces diagnostic errors, enhancing confidence in delivering accurate diagnoses. The technology aids in treatment planning by considering all relevant factors for patient‐centric decisions, improving outcomes. AI enhances care coordination by facilitating real‐time information sharing among providers. Additionally, continuous AI monitoring acts as a vital safety net, alerting providers to anomalies and supporting timely interventions. Overall, AI's role in preventive care and data analysis is seen as crucial for early intervention and patient well‐being (Table 4).

### 3.5 Ethical and regulatory aspects of AI

Nurse practitioners highlighted the critical importance of data security and privacy in AI use. They stressed the need for vigilance, encryption, and access controls to protect patient information, linking these measures to broader ethical and trust issues. Practitioners acknowledged the complexities of obtaining informed consent, emphasizing the need for comprehensive patient education and transparent dialogues about AI's role and implications. They also stressed the importance of addressing biases and ensuring equity in AI adoption to provide all patients with fair access and outcomes (Table 5).

### 3.6 Main theme: Education and training needs

Nurse practitioners advocated for integrating AI into health care education, emphasizing the need for comprehensive courses on AI fundamentals, applications, and ethics. They highlighted that including AI in curricula bridges theory and practice, equipping future professionals with essential skills. Practitioners called for tailored training programs that address real‐world AI applications and support professional development. They also recognized the challenges of keeping up with rapidly evolving AI technology, stressing the importance of continuous learning and institutional support to balance clinical duties with acquiring AI expertize (Table 5).

### 3.7 Collaboration and interdisciplinary relationships

In this research, nurse practitioners highlighted the benefits of collaborating with AI specialists, recognizing their technical expertize as vital for improving patient care. This partnership merges technical skills with a patient‐centered approach, introducing transformative tools that enhance health care delivery. Nurse practitioners also stressed their essential role in AI implementation, ensuring technology aligns with patient needs. Their involvement is crucial for developing patient‐centric AI solutions, thus aligning with health care's core mission to promote patient well‐being. This collaboration strategy is key to maximizing AI's benefits in health care (Table 6).

### 3.8 Obstacles in integrating AI

Nurse practitioners faced challenges integrating AI into their established systems, stressing the need for careful planning to avoid workflow disruptions. They emphasized the importance of seamless integration with existing practices to maintain a patient‐focused environment. Technical barriers and financial constraints were major concerns, with a strong emphasis on ensuring AI systems are compatible with current technology. Practitioners called for user‐friendly, cost‐effective solutions to enable smooth, financially sustainable AI adoption while minimizing disruptions to daily operations (Table 6).

### 3.9 AI and health care policy

Nurse practitioners emphasized the crucial role of flexible government regulations in health care AI. Collaboration between policymakers and health care professionals is vital to balance innovation and patient safety. Furthermore, they underscored the need for strong AI policies within health care institutions. These policies offer clear ethical guidelines, ensuring transparency, trust, and adaptability in the face of evolving technology. In addition, nurse practitioners highlighted the significance of equitable reimbursement policies for ethical AI integration in health care. These policies should provide incentives for AI usage, align with ethical principles, and ultimately enhance patient care and outcomes (Table 6).

## 4 DISCUSSION

This study presents a nuanced view of nurse practitioners' perceptions and attitudes toward AI in health care, encompassing their perspectives on implementation, impact, ethics, education, and integration challenges. This discourse underscores the evolving relationship between health care professionals and AI, highlighting both the opportunities and obstacles that AI introduces into the health care domain.

A notable finding from the study is the shift in nurse practitioners’ attitudes towards AI, especially in its diagnostic role. They recognize AI's potential to process vast amounts of patient data and identify patterns that can enhance diagnostic accuracy. This aligns with existing research indicating that AI complements, rather than replaces, clinical judgment.^35^, ^65^ The growing acceptance of AI in diagnostics reflects a broader trend towards integrating AI into health care decision‐making processes.^66^, ^67^ Nurse practitioners increasingly view AI as a valuable asset in treatment decision‐making, offering personalized recommendations based on patient‐specific data. This acceptance is supported by other studies demonstrating AI's ability to improve treatment quality through evidence‐based guidance^68^, ^69^ and its integration into clinical decision support systems.^70^

AI's role extends to facilitating nursing care planning by analyzing patient data to predict complications and suggest individualized interventions.^71^ This capability is seen as a significant enhancement to personalized care and effective management of complex cases. In patient communication, AI is viewed as a virtual health companion that can provide information, answer questions, and support patient education. This perspective is supported by research highlighting AI's potential to improve patient engagement and education.^72^, ^73^

The study also reveals that nurse practitioners appreciate AI's potential to streamline workflows and reduce administrative burdens, aligning with studies that demonstrate AI's effectiveness in optimizing health care processes.^74^, ^75^ Additionally, AI‐driven predictive analytics are recognized for their potential to improve patient care outcomes through data‐driven insights.^28^ However, despite these positive aspects, nurse practitioners' express concerns about the dehumanization of care, job displacement, and bias associated with AI. These concerns echo the broader apprehensions about AI in health care, emphasizing the need for responsible AI integration that complements rather than replaces human roles.^38^, ^76^, ^77^

The balance between technology and human interaction is a key theme. Nurse practitioners emphasize the importance of maintaining a personal connection with patients while using AI as a supportive tool, aligning with literature on preserving the human touch amidst technological advancements.^78^, ^79^ Effective communication about AI's role is crucial for maintaining transparency and patient‐centered care.^80^, ^81^

The study also highlights AI's role in improving health care delivery, with nurse practitioners recognizing its impact on reducing diagnostic errors, enhancing treatment planning, and improving care coordination. This is consistent with research affirming AI's potential to enhance efficiency and safety in health care.^82^, ^83^ AI's continuous monitoring capabilities are praised for their role in patient safety,^84^, ^85^ and its potential in preventive care by identifying risk factors is also noted.

Ethical and regulatory considerations are paramount. Nurse practitioners emphasize the importance of data security, privacy, and informed consent in AI use, aligning with the broader emphasis on protecting patient information.^86^ They advocate for comprehensive education and training on AI, noting the need for up‐to‐date courses covering AI fundamentals, applications, and ethics.^87^, ^88^ Collaboration between nurse practitioners and AI specialists is seen as vital for enhancing decision‐making and patient care, stressing the need for interdisciplinary approaches.^89^, ^90^

The study also addresses obstacles to AI integration, such as workflow disruptions, technical barriers, and financial constraints. Nurse practitioners call for meticulous planning and cost‐effective solutions to facilitate smooth transitions. Furthermore, they highlight the need for flexible government regulations and ethical guidelines to ensure responsible AI integration in health care institutions.^91^

## 5 IMPLICATIONS OF THIS STUDY

This study has significant implications for health care. It reveals that nurse practitioners' views on AI integration are varied, with some enthusiastic and others concerned. This calls for tailored strategies to educate and engage practitioners on AI's benefits and risks. Addressing their concerns about AI exacerbating health care inequality is crucial; AI systems must be designed to ensure equitable access and quality. The study also highlights the need for robust regulations to safeguard patient privacy, data security, and informed consent in AI use. Clear regulations will help build trust in AI systems. Finally, nurse practitioners are vital for maintaining patient connections and explaining AI use. They will need specialized training to effectively balance technology with personal care and support patients throughout their health care journey.

## 6 LIMITATIONS OF THIS STUDY

This study on nurse practitioners' perceptions of AI in health care has several limitations. First, its geographical focus on Dhaka, Bangladesh, may limit generalizability to other regions with different health care systems and cultures. The purposive sampling method, while ensuring diversity, may introduce bias due to specific inclusion criteria, such as prior AI experience or conference attendance, potentially skewing perspectives. Additionally, relying solely on semi‐structured interviews may miss insights that surveys or observations could provide. The study also did not explore the impact of the COVID‐19 pandemic on attitudes toward AI adoption. Furthermore, it did not fully address challenges related to AI implementation, such as data privacy, algorithm bias, and regulatory issues.

## 7 RECOMMENDATIONS FOR FUTURE RESEARCH

Future research on nurse practitioners' views on AI in health care should include several key areas. Longitudinal studies are needed to track how perceptions and attitudes evolve as AI becomes integral to practice, identifying trends, barriers, and opportunities. Exploring ethical issues, such as privacy, consent, transparency, and fairness, is essential to understand how practitioners navigate these dilemmas. Quantitative research should assess AI's impact on clinical decision‐making, diagnostic accuracy, and patient outcomes. Investigating how AI can be tailored to meet patient preferences and values is crucial for maintaining patient‐centered care. Evaluating the effectiveness of AI education and training programs will gauge their impact on practitioners' readiness and skill levels. Additionally, research should focus on overcoming challenges related to AI integration, including workflow disruptions, technical issues, and financial constraints. Finally, examining the influence of government regulations and institutional policies will help identify necessary adjustments for responsible AI adoption.

## 8 CONCLUSIONS

The study on nurse practitioners' perceptions of AI adoption in health care reveals a complex array of opinions and concerns. Key themes include AI's role as a valuable tool for diagnosis and treatment, but with mixed feelings about its impact on workflow, predictive analytics, and patient outcomes. Trust in AI decision support is crucial, necessitating confidence‐building among health care professionals. Attitudes vary, with concerns about health care inequality and regulatory compliance. Ethical considerations, such as privacy and data security, also influence willingness to adopt AI. The study highlights the need for a patient‐centered approach, effective communication, and overcoming technical and financial barriers. Education and training in AI are essential, with a call to integrate AI into educational programs. Health care policy plays a pivotal role in AI adoption, influencing regulations, insurance, and reimbursement. Overall, a comprehensive approach is needed to address diverse challenges and enhance health care quality while considering ethical and regulatory concerns.

## AUTHOR CONTRIBUTIONS

**Moustaq Karim Khan Rony:** Conceptualization, data curation, formal analysis, investigation, methodology, project administration, resources, software, supervision, validation, visualization, writing—original draft, writing—review and editing. **Sharker Md. Numan:** Conceptualization, software, validation, writing— review and editing. **Fateha tuj Johra:** Data curation, investigation, project administration, visualization. **Khadiza Akter:** Conceptualization, resources, supervision, writing—review and editing. **Fazila Akter:** Formal analysis, methodology, resources. **Mitun Debnath:** Data curation, resources, validation, writing—review and editing. **Sujit Mondal:** Formal analysis, investigation, writing—original draft. **Md. Wahiduzzaman:** Data curation, formal analysis, writing—original draft. **Mousumi Das:** Conceptualization, investigation, supervision, writing—review and editing. **Mohammad Ullah:** Conceptualization, project administration, visualization, writing—review and editing. **Mohammad Habibur Rahman:** Data curation, investigation, project administration, supervision. **Shuvashish Das Bala:** Data curation, resources, writing—original draft. **Mst. Rina Parvin:** Conceptualization, methodology, supervision, writing—original draft. All authors have read and approved the final version of the manuscript, and the corresponding author had full access to all of the data in this study and takes complete responsibility for the integrity of the data and the accuracy of the data analysis.

## CONFLICT OF INTEREST STATEMENT

The authors declare no conflict of interest.

## TRANSPARENCY STATEMENT

The lead author Moustaq Karim Khan Rony affirms that this manuscript is an honest, accurate, and transparent account of the study being reported; that no important aspects of the study have been omitted; and that any discrepancies from the study as planned (and, if relevant, registered) have been explained.

## Data Availability

The data that support the findings of this study are available on request from the corresponding author. The data are not publicly available due to privacy or ethical restrictions.
